# Book Reviews

**Published:** 1827-04

**Authors:** 


### CRITICAL ANALYSES.

Quæ laudanda forent, et quæ culpanda, vicissim

Illa, prius, cretâ; mox hæc, carbone, *notamus.*—Persius.

*Outlines of Human Physiology.* By Herbert Mayo, Surgeon, and Lecturer on Anatomy.—8vo. pp. 406, London, 1827.

During the rapid progress which the science of physiology has recently made, this country has not, perhaps, been behind her continental neighbours in the share she has contributed to its advancement; but it is somewhat remarkable that, till lately, not one original treatise upon this subject should have been written by an Englishman, and that the only works in the hands of our students have been translations of different foreign writers, particularly of Blumenbach and Richerand, whose works have been published by various successive editors: in several instances, indeed, with the addition of some very valuable and interesting notes and comments upon the text of the original authors. But there have been so many successful labourers in the pursuit of physiological knowledge, that the whole science, within the last few years, has assumed a new aspect: the most cautious deductions from experimental research are alone sufficient to satisfy the philosophic scepticism of modern science; and experiment has elicited so many important facts, that, in the translations alluded to, notes are required to explain even the comments themselves. Such a mode of publication, it must be obvious, will therefore be little adapted to the necessities of one desirous of acquiring any branch of science; some previous knowledge being necessary to enable the reader to compare the text and the notes together, and decide to which authority he is to give credence.

With this impression upon our minds, it was not without satisfaction that we anticipated an original English work from the pen of so distinguished a physiologist as Mr. Mayo had proved himself to be by his former publications. Nor have our expectations been disappointed; and we can now congratulate the medical profession on possessing a work which is well calculated to facilitate their studies.

It is indeed not very long since we had occasion to notice the publication of a work on Physiology by Dr. Bostock, which might appear to some of our readers to supersede the necessity for another publication on the same subject. But excellent as the two volumes which Dr. Bostock has yet published must be acknowledged to be, they will suit a part only, and that a small part, of those for whom a work on physiology was required. The labour and research which have been bestowed upon the compilation of his work, and the clearness and ability with which his extensive learning is brought forward, are calculated to reflect most deservedly the greatest credit upon his name as an author; and, to a reader who is already a physiologist, his volumes are themselves a library. There is not an author of the least celebrity whose opinions are not canvassed with the utmost ingenuity and candour; but, after reading the voluminous compilation, the student rises from the perusal of the numerous theories and experiments adduced upon any particular subject, at a loss to know by whose opinion he is to be guided; unable to ascertain which author Dr. Bostock is himself inclined to follow; and regretting, perhaps, that the learned compiler is not himself a practical physiologist.

The work which we now bring under the notice of our readers is free from the disadvantages to which we have just alluded. Its brevity does not allow of the discussion of the numberless hypotheses to which the science of physiology has given rise, but almost every part of it bears testimony to the labour which the author has himself bestowed on the various interesting inquiries he has undertaken to teach; and the clearness and total absence of unimportant matter which results from this practical knowledge of the subject, are such as to render the study easy and delightful to those who were previously completely ignorant of physiology. It is therefore adapted to every class of readers, and we venture to predict that in a short time it will become the text-book of the medical student, and will be studied by great numbers beyond the circle of the profession.

It is extremely difficult, in a review of a systematic work on any science, to make our readers acquainted with the real powers of the writer, or to give a complete knowledge of the subject. The brevity and conciseness, which are chief requisites in such a work, prevent any further abridgment. We shall only attempt, therefore, to lay before our readers an outline of the plan which the author has adopted, and to extract a few passages which may serve to convey an idea of the style and manner in which the work is conducted.

Mr. Mayo commences his System of Physiology by a few remarks on the tendency of physiological studies. He points out, in an eloquent passage, the first impression which the study of the functions of living beings is perhaps invariably calculated to produce,—viz. to raise the mind of the student from "nature up to nature's God;" and afterwards successfully combats the stigma so often thrown upon medical studies, that they lead to irreligion or materialism. The arguments commonly brought forward in support of the doctrine of materialism, are deduced from the intimate connexion which exists between the mind and the body, and from the analogy between the relation of thought to the brain, and that of other functions to other organs. It is unnecessary to illustrate the futility of the first argument we have adverted to; and, in regard to the second, Mr. Mayo has very clearly exposed a fallacy so frequently lost sight of, that we cannot forbear extracting his remarks.

"Physiologists have ascertained that different parts of the body have different offices; that the formation of bile takes place in the liver, of saliva in other glands; that a power of shortening belongs to certain fibrous parts; that consciousness is connected with the nervous system. Now, as the liver is admitted to be necessary for the separation of bile from the blood,—as the power of shortening is the result of the structure of a muscle, it has been supposed to be an analogical inference, that thought is the produce of the brain.

"But the separation of one fluid from another, and the shortening of a fibre, are expressions which convey no meaning if we attempt to abstract them from the notion of material organs or material substance. With the functions of the brain the case is different. Our conception of thought does not involve any attribute of matter; and we are struck with no speculative absurdity in supposing that our consciousness may survive after every material element, with which it has been connected, shall have perished. Thus the analogy is destroyed between the dependence of thought upon the brain, and that of other functions upon other organs; and the argument founded upon it falls to the ground." (P. 5.)

The remainder of the first chapter is devoted to a consideration of those properties which characterise organised matter, and the differences between the vital phenomena of vegetable and animal life. The properties of life are thus summed up by our author:

"Upon a strict analysis of the phenomena above enumerated, two properties, to which the whole appear referable, admit of being indistinctly shadowed out.

"1. The change wrought upon the ingesta during assimilation, which appears strictly analogous to some effects of chemical attraction, may be ascribed to a principle of vital affinity. In order to give this term an equal value with the term gravity, physiologists have only to determine with precision the physical conditions which invariably precede changes in the chemical nature of the ingesta, and of the component elements of a living body. It is highly probable that the laws of chemistry, and the property which controls the affinities of matter in living bodies, will prove eventually to be identical.

"2. The assumption of foreign matter, and its propulsion through the tubes of a living body, may be partially produced or promoted by capillary attraction, by elasticity, by impulse communicated from without, by gravity; but in the majority of instances another principle is distinctly in operation. It is ascertained that various parts in animals and plants alternately contract and expand when alive; or at one time have a tendency to become shorter in one direction, at another to extend themselves to their former dimensions: this property is termed Irritability. The fluids in the higher animals are thus set in motion by the contraction of the vessels which contain them. In the propulsion of the sap of plants, many phenomena prove that the same contrivance is used. It is analogically probable, that in all living bodies a like principle is employed upon this object. It remains for physiologists to determine the exact conditions under which different irritable parts contract or are relaxed." (P. 15.)

"When animal life begins, new properties are added. The polype, which in material organisation is infinitely more simple than the higher plants, gives proofs of sensation, instinct, and volition; yet, when divided, each half grows into a perfect polype. In the ascending scale of animals, the phenomena of consciousness are more and more developed; the structure of the body becomes proportionably more elaborate; the animal becomes individualised; its separate portions become incapable of independent existence; it consists of a single series of organs, the functions of which exert a reciprocal influence, and combine to sustain life." (P. 17.)

In giving this simple but accurate account of the vital properties, we could have wished that Mr. Mayo had not employed the term "irritability," since it has been used by different authors in such various acceptations as to lead to considerable obscurity in physiological reasoning. We observe that many parts of living bodies have the power of contracting in one dimension, upon the application of appropriate stimuli: all substances possessing a power of contraction beyond mere elasticity, are said to be irritable, and the property itself is called by many physiologists Irritability, in which sense the term is employed by Mr. Mayo. In this extended sense of the term, we shall find "that the parts of the human frame which possess irritability are muscular substance, the substance of the uterus, the fibrous coat of arteries, the unattached margin of the iris, some parts of the skin, and perhaps the dense texture which is employed in forming excretory tubes." (P. 35.)

But the term irritability is generally used by other physiologists to designate that kind of contraction only which can be excited by mechanical stimuli, and which occurs exclusively in muscular structure. Thus BLUMENBACH remarks, "Irritability (we mean the irritability of Haller,) is peculiar to the muscles, and may be called the *vis muscularis*." (Translation by ELLIOTSON, p. 18.)

Restricting the term to the power of shortening in muscular substance, there remain a variety of parts of the body, not muscular, and yet endowed with the power of contraction. Authors who use the term irritability in this limited sense, admit of a specific power of contraction in other textures, distinct from the irritability of muscles. Blumenbach even specifies a third kind of action possessed by some parts of living bodies, the various modifications of which he calls "*vitæ propriæ*;" to which head he refers the motions of the iris, the erection of the nipple, and the action of some other parts.

Muscular structure being irritable, we find further that irritability and muscularity are often used as synonymous terms; and thence arises another source of vagueness and obscurity in physiological language. Muscles are fibrous,—the middle coat of arteries is fibrous: but is it therefore muscular? Judging from analogy, we should answer in the negative, since a muscular coat is in some animals added to the common fibrous tunic. Is it irritable? No contraction ensues on pinching the nerves going to the vessels; and it is at least a disputed point whether any mechanical stimuli are capable of producing the motion of arteries: yet, in medical writings, the action of arteries is constantly spoken of as identically the same with that of muscle. Still less can the power which the vessels of plants possess of propelling the sap, be compared with the irritability "peculiar to the muscles."

We should wish, then, to see the term irritability, in the comprehensive sense attached to it by Mr. Mayo, changed for that used by other authors to designate the same property,—viz. Contractility, which seems to us to be much less liable to be used in different acceptations. Contractility will thus become a generic term, including all motion in living bodies which is effected by a peculiar vital property.

We shall in this manner be enabled to distinguish such bodies as are simply *contractile*, (in which class will be included the vessels of plants, as well as many parts of animals,) from those which are *contractile* and *fibrous* also,—such as the arteries of animals; while, again, we shall find another kind of animal substance which possesses both these peculiarities, which enjoys the property of *contractility*, which is *fibrous*, and which is also *muscular*. All are endowed with the power of contraction,—i. e. they are all irritable in Mr. Mayo's

Contractility — Expandibility acceptation of the word; but the last only possesses irritability, in the limited sense in which the term is used by other physiologists.

Having pointed out the peculiarities of living beings generally, Mr. Mayo next proceeds to describe the fluid from which all animal bodies are formed. Accordingly we find in the five next chapters an account of the blood, and of the different phenomena of the circulation; including a dissertation on muscular action,—on the forces which circulate the blood,—on the pulmonary circulation and phenomena of respiration,—and on the circulation through the body; in which last chapter the process of secretion is minutely analysed. Some of the subjects which are here described have engrossed so large a portion of the public attention during the last few months, that we are tempted to dwell longer on this part of the work than we should otherwise be induced to do; for we cannot but suspect that more interest has been excited by the ingenious investigations of Dr. Barry, than either their originality or intrinsic importance really entitles them to.

It has been long known that the mechanism of respiration exerts a considerable influence upon the transmission of the blood: this is proved by the turgid state of the veins of the neck during expiration,—by the motion of the brain corresponding with the state of the respiration,—by the occurrence of death in some instances where air has been drawn into a divided vein, through the power of suction exerted by the chest. The recent experiments of Dr. Barry have placed these phenomena in a still more striking point of view; but are we therefore justified in attributing the entire phenomena of circulation and absorption to the influence of this power? No doubt can be entertained, we imagine, that the circulation is assisted by the mechanical influence of atmospheric pressure; but we certainly believe that it is at best only an accessary power, and probably the action of the heart, and that of the arteries, and the resilience of the lungs, are each of them of more importance in the phenomena of circulation; and in many cases atmospheric pressure seems to be wholly out of the question. Thus the empty state of the arteries after death has been clearly shown by Dr. Carson to be owing to the resilient power of the lungs; and it is remarked by Mr. Mayo, that in the fœtal state, and when the circulation is kept up, after laying open the chest, by artificial respiration, that the spontaneous dilatation of the auricles is the only power which can promote the entrance of the blood into those cavities, independent of the vis a tergo communicated by the ventricle and by the action of the arteries. In the latter case, however, may not the elasticity of the lungs exert some influence, so as to interfere in some measure with the accuracy of this conclusion? A curious case, indeed, is extracted by our author from an account published by Mr. Brodie in the Philosophical Transactions, in which no heart was present in an imperfect human fœtus; but the umbilical vein communicated with the vena cava, which was distributed in the usual manner; while the umbilical artery terminated in the left internal iliac, which ended in an aorta having no arch, but only branches which supplied the head and arms, there being no communication between the trunks of the arterial and venous systems.

"In this and similar cases, it is presumed that motion, limited to one direction by the valves in the venous system, is given to the blood by the contraction of the arteries and of the capillary vessels. Upon a like supposition, the fact has been explained that, after the removal of the heart, if transparent parts of the body be examined in a microscope, the blood is seen to flow for a time in the capillary vessels." (P. 74.)

The following is Mr. Mayo's opinion of the influence of the chest upon the circulation. Speaking of Dr. Barry's experiment, in which a coloured fluid was made to rise during inspiration in a tube inserted into the jugular vein of a horse, he observes—

"It remains problematical how far this phenomenon be independent of the resilience of the lungs. For if we admit that, were the trachea closed, and the chest to be dilated, the atmospheric pressure being taken off the heart, and operating without resistance upon the veins of other parts, would drive their contents towards the right auricle; yet in natural breathing the trachea is open, and permits the air to enter the lungs as promptly as the chest enlarges: so promptly, perhaps, as, but for the resilience of the lungs, to keep up a pressure upon the outer surface of the heart, equal to that which the air exerts through the column of blood in the veins upon its inner surface." (P. 66.)

To the other question of the influence of atmospheric pressure upon the absorption of fluids, the same remarks are applicable. Whatever power the elevation of the chest will exert upon the circulation of the blood through the veins, must operate in a proportionate degree upon the absorbents which communicate with the veins. The action of the cupping glass in preventing the absorption of poison, (a practical experiment of considerable importance,) is, we believe, more generally acknowledged to be produced by pressure alone upon the vessels, and not by the removal of atmospheric pressure from the mouths of the absorbent vessels, as Dr.

Barry at first imagined. It is no doubt precisely the same as that of a ligature, which has long been known to be attended with this beneficial effect. Recent experiments have proved, we believe, that a simple ring pressed around the place where poison has been inserted, is equally effectual in preventing the entrance of the poison into the system, as when an exhausted cupping-glass is placed over the puncture. And an experiment alluded to by our author in another part of the work is conclusive upon the question, and proves decidedly that absorption will go on, in the lacteals at least, after all influence of the chest has been entirely removed by interrupting the course of the chyle towards the veins by a ligature.

We will pass over the next chapter, therefore, in which an account is given of the function of Digestion, and make the following extract from Chapter VIII. in which is explained the mechanism by which the Chyle, the product of digestion, is carried into the circulation.

"Thus it appears that the lacteal system originates by numerous capillary orifices upon the villi of the small intestines; and we may presume that the absorption of chyle commences upon physical principles. Accordingly, if the mesentery be exposed immediately after the death of an animal killed during digestion, and the contents of a lacteal be pressed forwards towards the thoracic duct, and a ligature be tied upon the empty vessel, the lacteal is found to become filled again with chyle by the continuance of intestinal absorption. By capillary attraction, the fluid with which it is bathed would ascend in the capillary orifice of a lacteal; and, if it rose beyond a single pair of valves, the contraction of the vessel itself would be sufficient to urge it onward to the venous system." (P. 183.)

Mr. Mayo is inclined to side with M. MAGENDIE in believing that the lacteals absorb chyle only from the intestines, and points out the following probable source of fallacy in HUNTER's experiment, in which a solution of starch and indigo was placed in the cavity of the intestine. In repeating the experiment upon a rabbit, he observed—

"The lacteals, which, when a solution of starch and indigo was first placed in the bowel, were full of chyle, on being examined half an hour afterwards, appeared of a clear blue colour, and those present were for an instant satisfied that the experiment had succeeded; but, upon placing a sheet of white paper behind the mesentery, the blue tinge disappeared,—the vessels were seen to be transparent and empty. On removing the white paper, they reassumed their blue colour." Again—"When Mr. Hunter saw a white fluid rise in the lacteals, after pouring milk into the bowel, we must suppose that some remains of chyle in the small intestine continued to be absorbed." (P. 184.)

As the lacteals evidently possess the power of absorbing chyle, it seems probable, from the similarity of the lymphatics in other parts of the body to the lacteals in the mesentery, that these vessels also are endowed with the property of removing substances presented in a fluid state to their extremities. Such has accordingly been the common opinion, from the time that the lymphatics were first discovered till the last few years, when some curious experiments of Magendie seemed to prove that the power of absorption may belong to other vessels; and many of his partisans, not contented with claiming this property of absorption for the veins, have chosen to carry their opinions so far as even to deny that absorption is at all performed by the lymphatic system.

Mr. Mayo does not go farther than the expression of his belief that "we are at liberty to conjecture, upon analogy," that the function usually ascribed to the lymphatic vessels is actually enjoyed by them; and he remarks, that "this conjecture, at any rate, is the most rational which has been proposed as to the use of the lymphatic system, and is remarkably borne out by various circumstances in diseases." (P. 186.)

As the interesting question of Venous Absorption has also been much agitated of late, we are tempted to give our readers the opinion of Mr. Mayo upon this subject, who attributes many phenomena, usually alledged as those of absorption, to the occurrence of transudation or imbibition in the living subject, as well as in dead matter. The actual occurrence of this mechanical effect in living bodies was long denied, and was probably unknown to Magendie himself when he published his Experiments on the Absorption of Poison by the Veins. We recollect having somewhere seen an acknowledgment by Magendie himself, that, the existence of this property of living bodies being proved, his experiments are no longer conclusive of the absorption by the veins; though we believe, from the usual tenor of medical conversation upon this subject, that the phenomenon of transudation during life is very partially known. Mr. Mayo brings forward, however, at page 115, too many facts to leave any doubt upon our minds of the positive existence of this circumstance. M. Magendie's celebrated experiment, in which he insulated the crural artery and vein of a dog, is well known. M. SEGALAS performed another experiment, which, like Magendie's, clearly proved that poisons may be introduced into the system through the veins. He tied all the vessels of a portion of mesentery, except the lacteals and one large artery. A vein, punctured beyond the ligature, allowed the blood carried through the artery towards the intestine to escape. Some nux vomica was then placed in the intestine; but no symptoms of poison manifested themselves during an hour, although the lacteals remained free. The ligature was then removed from a vein, and in six minutes from this time the poison began to produce its usual symptoms. In speaking of these experiments, Mr. Mayo thus states his opinion upon the proofs which they afford of venous absorption.

"The preceding phenomena admit of two hypothetical solutions. We may suppose either that the veins possess a special power of absorption, through some mechanism not yet discovered, or that a poisonous substance may find its way through the coats of the vessels, by virtue of that sort of imbibition or transudation which takes place in dead matter, whether organised or unorganised, and which it is analogically probable takes place in living matter as well. The latter supposition has the recommendation of assuming nothing." (P. 115.)

"A popular objection to this view is founded upon the fact, that, upon opening the body of an animal immediately after death, the parts adjoining the gall-bladder are not tinged with bile. But it is easier to imagine that the bile is washed away by the circulating blood, or carried off by the lymphatics, as fast as it exudes, than to suppose a new principle in the living body competent to suspend the common law of imbibition by porous substances." (P. 119.)

This principle being established, the importance of the experiments is very much diminished, since they are no longer able to demonstrate that veins have the power of absorbing any substances presented to their extremities; restricting the term absorption to that peculiar operation already described as performed by the lacteals. Still less will the experiments prove that the property of absorption is not possessed by the lymphatic system. All that they are calculated to show is, that, if any substance gets into the blood, it may be carried into the centre of the circulation by the veins; but it has been shown that the poison may reach the cavity of the veins by transudation only. Nor must it be forgotten that it is probable, from comparative anatomy, that other communications exist between the lymphatics and the venous trunks, besides the principal union on each side of the neck.

Having thus described the means by which the chyle is formed, and the means by which it is conveyed into the blood, the two next chapters are devoted to the consideration of the Excretory Secretions of the Kidney and of the Skin. In the chapter on the Skin, besides an account of the perspiration, we find a description of the anatomy of the skin, and of the phenomena of vital heat; the other properties of the skin, as an organ of touch, being investigated in a subsequent part of the work.

We are presented with an elaborate account of the Brain and Nerves in Chapter XI. which is divided into five sections. The first of these is intended to point out such parts of the nervous system as are common to all animals,—viz. "a central organ consisting of two chords, one corresponding with either half of the body, upon which nodular masses are generally placed; and, secondly, of other chords or nerves derived from the central organ to the sentient surfaces, or contractile parts of the animal." (P. 208.) After which are described the common properties associated with the simplest as well as the highest forms of the nervous system, which are those of sensation, volition, and instinct.

Having in the second section given an anatomical description of the human nervous system, Mr. Mayo separates such parts as appear to be necessary to sensation, volition, and instinct, and which belong to man merely as an animal, from those which are connected with the higher faculties. The former are said to be the medulla oblongata and spinal cord; and accordingly, in the third section, many interesting experiments are related to prove that "these parts are likely to be the organs connected with these moral phenomena, which are shared by animals, from a starfish to man inclusively." (P. 232.)

In the two remaining sections, Mr. Mayo has given an account of the functions of the remaining part of the nervous system, namely the Brain; followed by some remarks upon sleep, dreaming, and sensorial illusions.

It is a much easier task, however, to discover such points of physical structure, and those moral phenomena which man possesses in common with all other animals, than it is to account, by any anatomical differences in the brain, for his intellectual superiority over the higher orders of the animal creation. The latter part of this chapter is, therefore, much less satisfactory than the preceding sections. Our limits will only allow of our extracting the following passage:

"But whatever vestiges we observe of human faculties in animals,—a susceptibility of attachment and of aversion, appetites like those of man, and a seeming sagacity in providing for their wants,—they yet exhibit nothing that can strictly be compared with human reason. Animals appear to share the same first impulses with human beings; but, not having the power to reflect, or deliberate, or conceive, the combination of means for the attainment of an end, they derive little advantage from experience, and want that capacity of indefinite improvement which alone might vindicate for man an exclusive claim to immortality." (P. 215.)

Say you know nothing about it.

The twelfth Chapter is devoted to an investigation of the five special senses, and is divided into as many sections. The first of these, on the Organs of Vision, is one of the most perfect and satisfactory discussions upon this extensive and difficult subject which we have yet read, and it appears to have received in an especial manner the attention of the author. We extract from it the following recapitulation of the offices performed by the retina, and which result from its form and situation.

"By retracing our steps from the point at which we have now arrived, we may attain, perhaps, yet clearer notions of the wonderful art displayed in the construction of the eye. The following different ends are seemingly comprehended in its design: 1. Each point of the retina being likely to receive the impression of rays of light at various angles, is so constituted as to refer visual impressions to one direction only, in order that the information conveyed to us may be consistent. 2. The retina is placed behind refractive media, which collect in one point upon its surface the rays that enter the eye from a given point in the object, in order that the same part of an object may not be seen in several places at once. 3. Each point of the retina has a definite inclination, in order that vision may be true; or, in other words, in order that the visual direction of an object may correspond with its tangible direction. 4. The retina is concave, in order to simplify the optical contrivances by which the cones of rays that enter the eye are brought to focal points upon it. 5. The retina being concave, the images of entire objects are necessarily reversed upon it to be seen in their true position; but the retina might have been convex, and then, for correct vision, the images of objects would have been delineated upon it erect." (P. 289.)

We next have, in the thirteenth Chapter, an account of the Human Voice, which is derived principally from M. Magendie's description. This is followed by a chapter on the Attitudes and Movements of Man, in which is included a history of the nature of Bone. The two concluding chapters are on Generation and on Growth; and, in discussing the latter subject, the author has introduced some original experiments of his own to illustrate the nature of the reunion of divided parts, some remarks upon which have already appeared in this Journal.

Such is a general view of the plan which Mr. Mayo has pursued in his System of Physiology. It is, perhaps, unfair to criticise the arrangement of an extensive science, where each function is so intimately connected with the rest; and the difficulty of forming any division which shall be entirely unobjectionable, is evident from the variety in the arrangement of the subject adopted by different writers on Physiology:

yet we are not satisfied that the plan followed by our author might not be materially improved. Neither does the execution of all parts of the work appear to us to be free from faults. We might point out particularly the latter part of the fifteenth chapter, relating to the development of the fœtus, as being evidently drawn up in a hasty and imperfect manner. We may also mention the section upon the Organ of Hearing, as an instance of two or three passages which are written in much too short a form to be easily intelligible to the student. These are faults, however, which can easily be corrected in a future edition; but we fear it would be more difficult to modify the arrangement so as entirely to please us. On the whole, however, it will be evident that we entertain a very high opinion of the work. The passages we have extracted may, perhaps, be sufficient to show, to a certain extent, the talent displayed by the author, and illustrate his usual style. It is clearly his design, in every part of the work, to condense his facts and reasonings into as small a space as possible; a design which is, perhaps, in some parts carried to the verge of a fault, as there are passages so closely written as to keep the mind too much on the stretch in order completely to follow the author's meaning. It is evidently the composition of a powerful and sagacious mind, and for the most part is couched in language proportionably terse and energetic; and this is most satisfactorily shown in those parts which require the greatest exertion of ingenuity and talent, such as the chapters on the Brain and on the Eye, which are distinguished by a degree of acuteness and logical precision very unusual in medical writings. Entertaining this opinion of Mr. Mayo's work, we most strongly recommend it to the perusal of our readers.

---

*Observations on the System of Teaching Clinical Medicine in the University of Edinburgh, with Suggestions for its Improvement; humbly submitted to the Consideration of the Patrons and Professors of that Institution.* By James Clark, M.D.—1827.

Dr. Clark is already favourably known to our readers as the author of an excellent little work on the Medical Topography and Statistics of the South of France and Italy, and as the spirited defender of British medicine, in two Italian Tracts published at Rome four or five years ago. The pamphlet before us, which we believe is only printed for private distribution, is on a subject of the greatest importance, not merely to the University of Edinburgh, but to every professional man who has at heart the interests and honour of the body to which he belongs. What the author says of Edinburgh, applies *a fortiori* to the London School, which can hardly be said to have even the semblance of clinical lectures. In a few of our hospitals, it is true, lectures are occasionally given; but there neither is, nor ever has been, in this great city a formal course of clinical medicine for the progressive instruction of the pupil. We hope this reproach is not to be perpetual, and we now call upon our hospital physicians to consult not only the interests of the profession, but their own personal fame and advantage, in instituting regular courses of clinical lectures.

Our readers will give us credit for sincerity when we say that we are none of those who run after every novelty of doctrine or practice that may be brought forward; and still less are we disposed to surrender the sterling and energetic practice of England, to the ultra-refinements of the present fashionable dogmas of the continental schools. At the same time, we have ourselves derived too much benefit from viewing the good and the evil of foreign practice,—and especially from the minute and unlimited inspection of bodies after death,—to doubt for a moment that the best of all schools for an observant and a sober-minded practitioner, is the wide school of continental Europe. At all events, it is only there where a just idea of what clinical medicine is, and what clinical instruction can effect, is to be obtained. It is only by observing better systems that we are led to discover the defects of our own; and we must consider the whole profession of this country under great obligations to Dr. Clark for making them acquainted with the very superior manner in which clinical medicine is taught in foreign countries. Well will it be for the University of Edinburgh if she listens to the voice of her son, and adopts some part, at least, of his friendly suggestions: and evil, we fear, if not shame and disgrace, will come upon her, if, too confident in former fame, she disregards these improvements which the spirit of the age demands, and on which alone she can securely rely for making the future equal to the past.

It appears that at this very time a royal commission is engaged in an inquiry into the state of education in the Scottish universities. It is this circumstance which has called forth the observations of Dr. Clark; and when we consider the earnestness and zeal, and at the same time the moderation and good sense, with which these are written, and, above all, the unanswerable reasons he adduces in favour of the proposed changes, we cannot help entertaining a confident hope that his pleading will have weight with those to whom it is addressed, and who have the power to make the requisite improvements.

"I would therefore, (says Dr. C.) in common with every member of the profession who has its real interests at heart, beseech you to consider well before the final arrangements are made,—to avail yourselves, to the full extent, of the fair opportunity which is now afforded of rendering the system of medical education in the University of Edinburgh equal, if not superior, to that of the best medical schools on the continent. It may be long before so favourable an occasion shall again offer; and, if the present inquiry is allowed to pass over without effecting material improvements in the course of medical education, the consequence will, in all probability, prove not a little injurious to the future interests and reputation of your school. Upon what is done now will depend the station which the medical school of Edinburgh is to hold, for the future, among similar institutions in this country; whether it is to stand pre-eminent as heretofore, or to be speedily surpassed by others. Recollect that what you do not may, and most likely will, be done by other medical schools, and Edinburgh will be ultimately compelled to follow in the march of improvement when she should have led; and may be reduced, perhaps, in consequence, to see her halls deserted in the department of science to which she has been indebted for her most extended celebrity."

Of the justice of the following remarks on the importance of clinical instruction, there can only be one opinion. We ourselves can bear mournful testimony as to how much time may be strenuously mis-spent in early attempts to study medical science, through a defective system, and owing to the want of a good clinical guide.

"Of the various branches of medical education, clinical instruction is, beyond all question, the most important, and is, in truth, that to which the others are merely preparatory and subservient. It is only at the bedside of the sick that the pupil, guided by an experienced practitioner, can make with advantage, or ought to make at all, his first acquaintance with diseases, or learn to observe them with the accuracy which they require. He may, indeed, have heard excellent descriptions of disease delivered in lecture-rooms, and read much of it in books; but the impressions he receives from such sources are faint and transitory, compared with those produced by actual intercourse with the sick. Personal observation, therefore, is absolutely necessary to the right investigation and conception of the phenomena of disease; but, in proportion as the first impressions produced by these are strong, is it of importance that the pupil's observation should be rightly directed. This can only be done by a well-conducted system of clinical instruction." (P. 3.)

"In the foreign universities, generally speaking, there is no part of the physician's education watched with more jealousy,—and there is no class of professors whose appointment excites more general interest than the clinical professors, or in whose selection and nomination greater care is taken that none but such as are well qualified for the office shall obtain it. In the case of no other professor, indeed, is there required a greater combination of the rarer qualities of the physician; and no one certainly has more important duties to perform. Upon his efforts, favoured by a judicious system, depends, in a great degree, the pupil's future progress in his profession. According to the direction which his mind receives during his clinical studies, is he, in all probability, to become an accurate observing physician, or an empirical writer of prescriptions. There can be little doubt that to a defective clinical education, more than to any thing else, is to be attributed the large proportion of routine practitioners in the profession,—of men who blindly follow the beaten track of their predecessors, without knowing or inquiring whether it is right or wrong; because, having never learned properly to observe disease for themselves, or to reason upon its phenomena, they can derive but little advantage from experience. For the truth of these remarks, I appeal to those members of the profession who have themselves suffered from a defective medical education, and who, after groping their way for years without deriving much advantage from their experience, have at length acquired the power of observing disease as it must be observed to be understood. How different is their future practice, both as regards their own improvement and their patients' welfare!—and how much more might they, as well as their patients, have profited by their early practice, had they enjoyed the advantages of a good clinical education! It is in a clinical course only, with the examples before him, that the professor can impress effectually upon the pupil's mind the necessity and the manner of observing minutely the symptoms of disease, in order to enable him to trace the connexion which exists between them and the morbid conditions of which they are the signs. And it is here only that the pupil can learn to distinguish the symptoms of disease from disease itself, or discriminate the signs which are merely indicative of functional derangement from those which are characteristic of organic change. Nor will the professor fail, as the examples offer, to point out the manner in which the phenomena of disease are modified by age and sex, by peculiarities of constitution, and by the existence of previous disease, either in the organ affected or in the neighbouring organs: neither will he omit to remark the effects which any epidemical influence reigning at the time may have in modifying the character of the diseases under treatment; as these are all circumstances which affect most materially the degree, the progress, and the issue of the disorder, and which require a corresponding modification of treatment. But above all, I repeat, it is his paramount duty to impress upon the pupil's mind the necessity of minute and comprehensive observation, as the surest protection against those wild theories and sweeping generalisations, which still continue to disfigure medical science, and which produce the most injurious effects on the minds of youth, by leading them aside from what I consider the only true path in which to attain sound practical knowledge." (P. 15—17.)

The following brief statement points out what our author considers defective in the present system of teaching clinical medicine in the University of Edinburgh. This is defective, he says—

"1st. Because the period of attendance required of the candidate for a medical degree, is not sufficiently long to admit of his acquiring such a share of practical knowledge as every graduate ought to possess.

"2d. Because one, or even two professors are not sufficient to teach clinical medicine efficiently to the number of medical pupils at present frequenting the University of Edinburgh.

"3d. Because the professor has too many patients to examine and prescribe for, during the time allotted to the clinical visit; which ought to have for its object the instruction of the pupil, as well as the treatment of the sick.

"4th. Because the clinical lectures are but few.

"5th. Because the pupils have little or no opportunity of acquiring any practical experience under the direction of the professor.

"6th. Because the clinical professor retains the charge of the clinical wards for too short a period at one time, and resumes it at too distant intervals." (P. 18.)

And he proposes to remedy these defects—

"1st. By increasing the period during which the candidate for a medical degree shall be required to attend to clinical medicine, (as, without sufficient time, he cannot possibly acquire a knowledge of practical medicine, however fair the opportunities held out to him may be).

"2dly. By increasing the number of clinical professors in office at the same time.

"3dly. By diminishing the number of patients under the charge of each professor.

"4thly. By increasing the number of clinical lectures.

"5thly. By making the office of clinical professor permanent, or fixed at least for a series of years, in the same individuals.

"And lastly. By instituting a practical clinic, upon the principle of the poly-clinics of Germany." (P. 19.)

We have not space at present to enter into all the details of these proposed amendments: we shall content ourselves with one or two extracts illustrating some of the principal points dwelt upon by our author. The following account of the German clinics is interesting: it is a modification of the system that Dr. C. proposes for the University of Edinburgh.

"There are two methods of teaching clinical medicine adopted in the medical schools of the continent, and two different kinds of clinical institutions. One of these may be called the *hospital clinic;* and the other, which is chiefly in use in Germany, is called there the *poly-clinic* or *ambulatory clinic.*

"In the hospital clinic, the elder pupils are appointed to attend to certain patients, of whom they may be said to have the charge, under the immediate inspection of the professor. They draw up the history of the cases under their care, and are examined on the nature of the disease by the professor, in the presence of the other pupils. They are also required to point out the symptoms which more especially characterise the disease, and serve to distinguish it from others which it most nearly resembles, and with which it might be confounded; and, finally, to give the prognosis and method of treatment. Those pupils continue to attend the same patients; and at every succeeding visit they examine, under the eye of the professor, the state of the symptoms, inquire into the effects of the remedies prescribed, &c. Of the whole case they keep a faithful record. If the disease should prove fatal, the attending pupil is further required to state, previously to the examination of the body, (a thing which is never omitted,) the morbid conditions which he expects to find, and what he considers to have been the cause of death." (P. 5, 6.)

"The poly-clinic of the German schools resembles closely our general dispensaries. As in them, the patients with chronic diseases, who are able to come abroad, are examined and prescribed for at the clinical rooms, and those labouring under acute diseases are seen and treated at their own houses.

"The pupil is first exercised in examining the patients that come to the clinic, under the observation of the professor, and is required to state the nature of the disease, its treatment, &c. as in the hospital clinic. The treatment being agreed upon, the pupil writes the prescription, which is examined, modified if necessary, and signed by the professor. After a time, the pupil is entrusted with the care of the out-patients. He is required to draw up an accurate history of each disease under his care, which is submitted to the inspection of the professor; as are also the reports of the progress and treatment of the case. Moreover, when he finds himself in difficulty, or the case appears to the professor to require it, the clinical assistant accompanies him to see the patient, and assists him with his advice. In urgent cases, I believe, the professor also visits the patient; and, where the disease proves fatal, he superintends the examination of the body; a practice to which, on the continent, objections are very rarely made." (P. 8.)

From the following account of the length of study imposed on the pupils of the continental schools, we cannot help turning, with something like shame, to consider the regulations that obtain in several of our British institutions. From this statement, it were well also if some other of our authorities, as well as Edinburgh, took a lesson. In common with all the well-wishers of science, we are sorry to observe that the dignity of our profession is daily wounded by unworthy members, who, envious of those above them, strive—not to raise themselves by honourable means—but to drag their more successful competitors down to their own level: and we know of no method so likely to avert this evil, as that of raising the standard of qualifications for those who present themselves for legal authority to exercise the respective branches of the profession. Sure we are, that degrees and licences are at present too much within the reach of ignorance and inexperience.

"Respecting the time, also, during which the pupil is required to attend to clinical medicine, the difference between the two systems is not less remarkable; and here, again, the advantage is greatly on the side of the foreign schools. According to the statuta of the Edinburgh University, six months' attendance on the clinical course, and the same length of time at any respectable hospital, is all that is required of the candidate for a medical degree. In the foreign universities, the period of attendance is much longer. At Pavia, which may be taken as an example of the practice at all the medical schools in the Austrian dominions, the pupil is required to attend the medical clinical course during four sessions, or two scholastic years. At the University of Turin, the same period is exacted; and at those of Paris and of Pisa, two full sessions. These may be taken as examples of the length of the clinical course at the continental medical schools. But this is not all: the period I have stated is requisite to qualify the pupils for taking the degree of doctor of medicine, but does not qualify them to practise. To obtain this privilege, they must have passed a considerable time, after graduation, in some hospital; or have attended the practice of a physician. The period prescribed in Austria, in Piedmont, and in Tuscany, is two years; and, in the latter country, the graduate of the University of Pisa must pass the two years of probation at Florence or Siena. After the completion of the period of probation, the graduate undergoes another examination on practical medicine, and must be approved of by his examiners, before he is admitted to the *libera praxis* of his profession. In Prussia, the period of probation is not so long; but the medical graduates, after having attained the degree of doctor of medicine in any of the universities of the kingdom, are obliged to pass some months at Berlin, when the schools are in activity; and are required to give proofs of their acquaintance with practical medicine, before they are permitted to practise." (P. 12, 13.)

The present notice of our author's views and proposed improvements will meet many eyes beyond the circle to which the original paper must be confined; and we trust it will not meet every eye in vain. Strange, indeed, it will be if, among the hundreds of hospitals and dispensaries that flourish in this country, none shall be found ready to adopt improvements at once so great, so obvious, and so easy. We, however, augur better things; and we yet expect to see the day when clinical medicine shall be as well understood, and as justly appreciated in this country as in Germany, France, and Italy. Reforms of this kind shall always find in us staunch and zealous supporters, because they tend directly to improve and elevate the character of our profession; and because the motives in which they originate are as pure as the ends sought to be attained by them are beneficial. Here we have no fierce and unworthy passions secretly at work, struggling for vent like the imprisoned fires of the volcano, and striving to undermine and level with the ground all noble and venerable monuments of former times. Here we have no smooth-tongued backbiter, who stabs in secret the reputation he dares not openly to assail;—no furious and unprincipled incendiary who hopes, by the destruction of all protecting institutions, to build his fortune on the ruin of all that is dignified, respectable, and respected. In the little work before us, if we have the zeal of the reformer, we have also the good faith of the patriot, and the respect and affection of the son and pupil. The hand that uncovers the blemishes, does so with gentleness and reverence, and, like the surgeon, only does so to heal them. The ambition of the author seems to be akin to the spirit which, during the last twenty years, has been gradually transforming the rude and incommodious buildings of the old University of Edinburgh into one noble and splendid pile, worthy at once of the objects for which it is destined, and of the liberal taste of the age. He seems only anxious for the adoption of his proposed improvements, because he is convinced they will add to the strength and beauty of the moral fabric of his Alma Mater. In this opinion we entirely coincide; and we shall ever be proud to support and encourage those who come forward as the true friends of our antient and venerable seminaries of learning, convinced as we are that their welfare is essentially connected with the prosperity of the country.

*Répertoire général d'Anatomie et de Physiologie, Pathologiques, et de Clinique Chirurgicale; ou, Recueil de Mémoires et d'Observations sur la Chirurgie, et sur l'Anatomie et la Physiologie, considerées dans les tissus sains et les tissus malades.* No. III. —Paris, 1827.

The first paper in the present Number of the Repertoire is from the pen of M. Breschet, upon the subject of *Ectropium of the Organs of the Circulation, and particularly of the Heart.* As it does not bear upon points which are practically important, we shall give it but brief attention. M. Breschet observes, that the study of the preternatural arrangement of parts has developed many facts respecting the animal organisation, and has given a solution to many problems concerning organic evolution. It will be easy, he thinks, to show that many affections peculiar to early infancy are not accidental, but a consequence of some fault in the primitive organisation. Whenever the heart has been found wanting, there has always been detected great irregularity in the other parts of the circulating apparatus. Absence of the heart has scarcely ever been observed, excepting in fetuses of monstrous formation. The brain and spinal marrow have either been defective or entirely wanting. Acephalous monsters are generally without a heart. Breschet, Meckel, and Tiedemann, have each observed this fact. The more or less complete absence of the heart, with a regular distribution of the arteries and veins, affords one of the strongest arguments in opposition to the theory of Haller, concerning the original appearance and development of those organs. According to that great physiologist, the heart is the *punctum saliens, primum vivens,* and the vessels proceed from it.

A great variety of writers have described various malformations and preternatural positions of the heart: M. Breschet confines himself principally, in the present paper, to particular points which have escaped previous observation. Five cases are related of preternatural formation of the heart, brain, &c. which are further illustrated by plates in the accompanying Atlas. The paper concludes by a few general reflections and conclusions upon the subject of such malformations, which may be perused with some interest by those who are devoting their attention to occasional deviations from the common march of nature.

*Memoir upon the Original or Congenital Displacement of the Head of the Femur.* By Baron Dupuytren.—This distinguished surgeon observes, that there is a kind of displacement of the superior extremities of both femurs, which he cannot find mentioned by any author, although he has made extensive researches. The principal objects of the paper are to prevent practitioners from forming erroneous opinions of the nature of such cases, and to rescue patients from the useless and severe modes of treatment, which are sometimes thought necessary.

The displacement consists in a transposition of the head of the femur from the acetabulum into the external iliac fossa. It is seen from birth, and appears to result from a considerable defect in the acetabulum, rather than from any accident or disease. The displacement of the parts is similar to that which constitutes luxation of the femur upwards and outwards. Two kinds of luxation of the head of the femur are already known, accidental and consecutive, spontaneous or symptomatic. Baron Dupuytren applies the term of Original or Congenital Luxation to the species he describes, in order to distinguish it from accidental or spontaneous luxation of the bone. This luxation is characterised, like all those in which the head of the femur is carried upwards and outwards, by a shortened state of the affected limb; a rising of the head of the bone into the external iliac fossa; projection of the great trochanter; retraction of almost all the muscles of the upper part of the thigh towards the crista of the ilium, where they form, around the head of the femur, a sort of cone, the base of which is at the os ilium, and the apex at the great trochanter. The tuberosity of the ischium is almost denuded, in consequence of its being deprived of its muscular covering. The limb is rotated inwards, and consequently the heel and calf of the leg are thrown outwards, and the toes and the knee inwards. There is an obliquity of the thighs from above downwards, and from without inwards, which is greater in proportion as the subject is more advanced in age, and as the size of the pelvis is more ample; and from which direction of the limb results a disposition of the femurs to cross each other at their inferior part. The whole limb is also emaciated, particularly its superior part. Its motions are necessarily impeded. There is an evident want of proportion between the upper and lower extremities of the individual, which is very observable if he be examined in a standing posture. The trunk of the body is fully developed, while the inferior extremities are short and small, as if they belonged to an individual of smaller stature. The attitude of such persons is also very peculiar. The upper part of the trunk is thrown backwards; the vertebral column projects very much forwards; the pelvis is placed almost horizontally upon the thigh-bones, and the points of the feet only touch the ground. These circumstances evidently result from the transposition of the ilio-femoral articulation, and from the centre of motion being thrown more backwards than usual. When persons who are thus formed attempt to walk, they rest upon the toes, and incline the superior part of the trunk towards the limb which is to support the weight of the body. The foot of the opposite side is raised from the ground, and the weight of the body transferred with evident difficulty from one side to the other; and each time that this is effected, the head of the femur which receives the weight of the body may be distinctly seen to rise into the external iliac fossa, the pelvis sinks down, and all the characters of the displacement become more apparent on this side; while on the other they are much diminished, until the moment when the limb receives, in its turn, the superincumbent weight: the effects of the displacement are then strongly marked, and are proportionably diminished on the opposite side. The cause of the painful and laborious efforts in walking is from the head of the femurs not being fixed, and from their being continually displaced. They are alternatively elevated and depressed, according as they have to bear the weight of the body, or as they are relieved from it. It will be easily conceived that so painful a locomotion prevents such individuals from going long distances.

In an horizontal position, the effects of this malformation are less visible, and in this position of the body the affected limbs may be shortened or lengthened at pleasure. To lengthen them it is only necessary to apply a slight extending force upon the extremity of the femurs, while they are shortened by being pushed towards the pelvis; and, if we compare the crista of the ilia and the top of the trochanters, it will be evident that, in these experiments, the heads of the femurs undergo a displacement of from one to two, or even to three inches, according to the age, the size, and the constitution of the individual. The position of the parts may be thus altered with the greatest facility, and without the production of the slightest pain. It is clear, then, that no disease can be present, and that the heads of the bones are not furnished with the natural cavities for their reception.

The diagnosis of this species of luxation is particularly important: it presents all the symptoms of that which results from disease of the articulation, and it has always been confounded with it, and, by an inevitable consequence, has been submitted to the same treatment, although it constitutes only a vicious formation of parts. In consequence of this error in the diagnosis, Baron Dupuytren has known some patients confined to their beds for several years with this original luxation, and others obliged to submit to leeching, blistering, repeated applications of moxa, &c. &c. The absence of all pain, swelling, abscess, fistula, or cicatrix; the simultaneous existence of luxation on both sides; the history of the individuals affected with this malformation; the appearance of the first symptoms, from the moment the individual attempts to walk; the progressive development of those symptoms, in proportion as the superincumbent weight of the body is increased, are means of distinguishing this species of luxation, which so materially differs in its origin, nature, and treatment, from other kinds of displacement of the femur. One very important circumstance to be observed is, that both the hips present the same alteration of form, a fact which so rarely occurs in disease of the articulation of the joint, that it may almost be deemed characteristic of the peculiar kind of formation just described. The history of such persons will be found to corroborate the proofs above adduced. They declare they have never suffered pain in the joint, and, in short, that they have been free from that severe and painful train of symptoms which generally leads to spontaneous luxation of the femur. And if the history be attentively pursued, it will lead to more than a negative result: it will explain, in a very positive manner, the early symptoms, progress, and development of the effects of congenital luxation of the hip-joint. As it rarely happens that the attention of the parents is directed to the fact until the child begins to walk, the surgeon is seldom called in till that period. When the dimensions of the pelvis are increased, and the child undertakes longer and more fatiguing exercise, the mischief then becomes more apparent.

The cause and nature of the evil, however, are still undetected, even by most professional observers. Many erroneous opinions are suggested as to the original cause of the deformity. Amongst others, a scrofulous diathesis is frequently suspected; and it must be confessed that the leucophlegmatic and rickety appearance of such individuals would give some weight to the opinion, if Baron Dupuytren had not witnessed such a faulty conformation in infants of a totally different constitution, and in whom no appearance of disease was to be observed. Upon dissection of the parts, no signs of actual or previous disease are to be detected.

At a more advanced age, when each sex assumes its distinctive marks, the more rapid growth and extent of the pelvis in the female render the effects more evident than in the male. With but moderate attention, any doubts that may previously have existed will now be removed. Each time that the femurs have to support the weight of the body, they are *disarticulated,* if the expression may be permitted.

The opportunities of determining by dissection the nature of this singular species of luxation are very rare. Life is not endangered by such an infirmity, and consequently Baron Dupuytren has only been able to study it in a few individuals who have perished from accident, or disease quite independent of the affection of the hip. The appearance of the muscles around the joint is considerably altered. Some are remarkably developed, whilst others are diminished in size. The action of the former has been preserved; that of the latter has been interrupted; and those of which the motions have been the most impeded are reduced to a fibrous and yellowish state, and in them we search in vain for any trace of muscular fibre. The superior part of the femur preserves its natural form and dimensions, excepting the internal and anterior side of the head of the bone, which is not so round as usual, in consequence of the friction it has undergone against parts not organised to receive it. The acetabulum is entirely wanting, or presents only a slight osseous and irregular surface, in which no articular cartilage nor synovial capsule is to be seen. It is surrounded with firm cellular tissue, and covered by the muscles which are inserted in the lesser trochanter. In two or three subjects examined by the Baron, the round ligament was much lengthened and somewhat flattened, and worn in particular parts by the pressure and friction of the head of the femur, which is found lodged in a cavity very similar to that which is observed in cases of accidental luxation. This cavity is very superficial, and without a well-defined margin. It is situated in the external iliac fossa, above and behind the acetabulum. In short, the appearances are similar to those which are observed in cases in which spontaneous or accidental luxation has occurred; with this difference, however, that every circumstance marks a very distant period, and seems to indicate an original faulty disposition of the parts.

The question to determine is, what can have been the cause of such a displacement, without any observable disease, and without any accidental violence? May it be considered as the result of some diseased action of the fetus in utero, which was spontaneously cured before birth, or the effect of some violence by which the head of the femur had been thrown from the acetabulum, which had been obliterated, without disease, in consequence of being thus rendered useless, and without its natural employment? M. Breschet has imagined that the acetabulum, which is formed by the union of three bones, may have been thus imperfectly formed in consequence of some obstacle to the evolution of those bones. Baron Dupuytren offers a few observations upon each of the explanations he has submitted. The fetus in utero, it is well known, is subject to many diseases, which may terminate either by a spontaneous cure or by its death, before birth. It is possible, then, that a disease similar to that which leads to luxation of the femurs, might produce the above kind of malformation. There are many circumstances, however, which militate against such an event. In the first place, all the children in whom such a displacement of the hip-joints was observed were healthy at the moment of their birth, which would hardly have been the case if they had previously suffered from a disease of so severe a nature as to have produced luxation of the femurs; and, again, no swelling, abscesses, or fistulous openings have ever been detected. It would appear that the displacement must rather be attributed to some violence which had forced the head of the femur from the acetabulum. To explain the manner in which such violence may have been applied, Baron Dupuytren offers the following remarks, which he thinks renders the cause he has adduced more probable: The position of the inferior extremities of the fetus in utero is such, that the thighs are powerfully bent upon the belly. The heads of the femurs make a continual effort against the posterior and inferior part of the capsule of the articulation. No injurious effects may result from this circumstance, if the fetus is of a firm and robust constitution; but if, on the contrary, it is of a lax fibre, and the tissues offer less resistance, the parts may suffer displacement: the posterior and inferior part of the capsule of the joint yields, and allows the head of the femur to pass. Luxation, in fact, takes place. To understand the displacement of the bone upwards and outwards, it must be remembered that the most powerful muscles which surround the articulation of the hip constantly tend to draw the head of the bone in that direction, when once it has escaped from the acetabulum.

M. Breschet infers from his own investigation of the subject, and from the labours of other modern anatomists, upon the evolutions of the embryon and fetus, particularly with respect to the osseous system, that the points the last developed are those in which either cavities or eminences are subsequently to exist; and that this tardy completion of parts is especially the case where several bony parts are to be united.

The application of this suggestion to the subject in question will easily be conceived. Baron Dupuytren truly enough observes, that, provided we could remedy the evil he has described, we might easily console ourselves for our ignorance of its cause. Unfortunately, however, in such cases we can do but little. By extension of the inferior extremities, we may perhaps for a moment bring them to their natural length; but, as the head of the femur has no cavity prepared to receive it, the limb would of course be retracted as soon as the extending power was no longer applied. The evil may, perhaps, admit of alleviation by palliative treatment, although not of a radical cure. If we bear in mind the natural tendency which the heads of the femurs have to ascend towards the external iliac fossa, and that the cause of such disposition to rise is, first, the weight of the body, which continually is tending to press downwards the pelvis between the femurs, and, in the second place, the action of muscles surrounding the head of the bone, the indications by which we must be regulated in our palliative efforts will be evident. It will obviously be necessary to prevent, as much as possible, the weight of the body from bearing upon an articulation which is thus imperfectly formed, and also to prevent the muscles from acting upon the femur, which, in consequence of the malformation of the joint, is not kept in its situation, nor restrained from rising towards the external iliac fossa. Rest, then, is one of the first means of preventing the head of the femur from rising, as it sometimes does, even to the crista of the ilium; and the sitting posture, in which the weight of the parts above bears upon the tuberosities of the ischium, instead of upon the ilio-femoral articulation, will be the most advantageous. Such occupations, therefore, as may be compatible with the sitting posture must be recommended for those who are labouring under this affliction, if they should be compelled to earn their livelihood.

Baron Dupuytren recommends the daily use of the cold bath, for three or four minutes. He presumes that the effect produced will be to strengthen the parts surrounding the accidental articulation, and to restrain in some degree the tendency which the heads of the femurs have to rise. A belt should also be worn surrounding the pelvis, which should pass over the great trochanters, and maintain them in an invariable position, by which the constant vacillation of the body upon a joint without an articulating cavity may be restrained. The belt should be placed upon the narrow part of the pelvis, between the crista of the ilium and the trochanters. It should be about three or four fingers' breadth, according to the age and stature of the patient. It should also be stuffed with cotton or horse-hair, and covered with buckskin, that it may not injure the parts to which it is applied. On the internal part, and at the lower edge of the belt, must be made narrow and superficial hollows, to receive the trochanters, and to maintain them in their situation. It must be confined round the pelvis by means of buckles and straps, and kept constantly in the position in which it is first applied by broad straps passing under the thighs, stuffed and covered like the girdle itself, and a little hollowed opposite the tuberosities of the ischia. Baron Dupuytren does not profess to have relieved entirely by these means the inconveniences of congenital luxation of the femurs: they have, however, been relieved in a certain degree. As a proof of the advantages of the belt applied in the above manner, he observes, that some patients, who became tired of wearing it, were again obliged to make use of it, as without it they had no firmness in the hips, nor could they walk with confidence.

In conclusion, the Baron remarks that congenital luxation of the femur is not so uncommon as may be supposed: he has seen twenty cases during the last eighteen years. He considers it worthy of observation that almost all the patients were females: not more than two or three were males out of the above number.

Three plates are given in the Atlas, representing, in three different aspects, the trunk of a young person of twelve years of age, affected with congenital displacement of the femurs.

We have dwelt so long upon the novel and very interesting discussion of Baron Dupuytren, that we can but mention the titles of the two next papers. The first is a brief Memoir by MM. H. Breschet and H. M. Edwards, describing some *Experimental Researches upon Pulmonary Exhalation;* and the second by M. A. Lembert, upon *Enteroraphia.*

